# Long-Term Follow-Up Regarding Pain Relief, Fertility, and Re-Operation after Surgery for Deep Endometriosis

**DOI:** 10.3390/jcm13175039

**Published:** 2024-08-25

**Authors:** Alexander Drechsel-Grau, Marcel Grube, Felix Neis, Birgitt Schoenfisch, Stefan Kommoss, Katharina Rall, Sara Y. Brucker, Bernhard Kraemer, Juergen Andress

**Affiliations:** 1 Department of Gynecology and Obstetrics, University Hospital Tuebingen, Calwerstrasse 7, 72076 Tuebingen, Germany; 2 Department of Urology, Cantonal Hospital St. Gallen, Rorschacher Strasse 95, 9000 St. Gallen, Switzerland; 3 Department of Gynecology and Obstetrics, Diakonie Klinikum Schwaebisch Hall, Diakoniestrasse 10, 74523 Schwaebisch Hall, Germany

**Keywords:** deep endometriosis, surgery, pain relief, fertility, re-operation, follow-up

## Abstract

**Background:** Endometriosis is known to be a common chronic disease that often affects the quality of life of patients. Especially for deep endometriosis (DE), the most challenging form of the disease, surgery remains an important component of treatment. However, long-term outcomes after surgery are poorly studied. Therefore, we aimed to evaluate the postoperative clinical course of women with DE who underwent surgery, particularly with regard to pain relief, fertility, and re-operations. **Methods:** Thus, women who underwent surgical treatment for DE between 2005 and 2015 were included in this retrospective questionnaire-based analysis. **Results:** A total of 87.0% of the patients who underwent surgery for pain reported a postoperative relief of their complaints. Moreover, 44.6% even stated that they were free of pain at the time of the questionnaire. Patients who underwent surgery for infertility and tried to become pregnant postoperatively gave birth to a child in 45.9% of cases. Approximately one-third of the patients had to undergo another surgery because of endometriosis-related symptoms. The main reasons for re-operation were pain and infertility. The median time to re-operation was 2.1 years. **Conclusions:** In this extraordinarily long follow-up with a remarkable response rate, we show that surgical treatment of DE leads to pain relief and improved fertility in most cases. However, the risk of recurrence and the need for re-operation remains remarkable.

## 1. Introduction

Endometriosis, defined as the presence of endometrial tissue outside the uterine cavity, is one of the most common gynecologic disorders with an estimated prevalence of 5% to 15% among women of childbearing age, although reliable data are lacking as the number of unreported cases may be substantial [1,2,3].

As it often causes different types of pain, such as dysmenorrhea, dyspareunia, etc., and is associated with infertility, the quality of life is affected in many cases, and the psychological burden on patients is high. In addition, the economic impact is remarkable due to the chronic nature of the disease [4,5,6,7]. Therefore, the efficacy of therapeutic options should be evaluated and continuously improved.

A specific type of endometriosis is deep endometriosis (DE), defined by an infiltration depth of more than 5 mm, with an estimated prevalence of 1% to 2%, as robust data are also scarce [8,9,10].

Even if DE can be spread throughout not only the abdominal cavity, it can often be found in characteristic localizations such as uterosacral ligaments, rectovaginal septum, vesicouterine fold, as well as bowel or urinary bladder.

The therapeutic approach should be determined individually, taking into account the symptoms and expectations of the patient. Hormone-based pharmacological therapy is able to improve symptoms, and best results are achieved in combination with surgery [11,12,13]. However, when used as the sole therapy, pharmacological therapy carries the risk of failing to extensively control the disease, especially if it is discontinued. Furthermore, side effects may force patients to discontinue pharmacological treatment, and negative effects resulting from long-term therapy must be considered. In addition, hormonal treatment cannot be used for women with a current desire to have children [14,15].

Therefore, surgery remains an important element in the treatment of symptomatic DE in most cases. Furthermore, when organ function is likely to be lost, surgical treatment is the only way to prevent organ failure (e.g., hydro-nephrosis caused by endometriosis affecting the ureter).

In most cases, surgery is performed minimally invasively due to reduced morbidity compared to open surgery. For best results regarding the improvement of clinical symptoms, complete resection of DE should be the aim of any surgery, taking into account possible intra- and postoperative complications [16,17,18,19,20,21,22].

For resection of DE, the surgical spectrum needed is complex and includes procedures such as ureterolysis, neurolysis, or preparation of the rectovaginal space. Therefore, patients should be referred to a tertiary endometriosis center for surgery.

However, the extent of surgery correlates with the risk of complications, especially if bowel surgery needs to be performed [22,23,24,25].

In our retrospective analysis, we evaluated the efficacy of surgery for DE, especially in terms of improvement of symptoms, fertility, and the need for re-operation, by examining the postoperative clinical course of patients.

## 2. Materials and Methods

Patients who underwent surgery for treatment of DE at the highest-level endometriosis center of the Department of Women’s Health Tuebingen between 2005 and 2015 were included in this retrospective study. DE had to be diagnosed according to at least one of the following criteria: Histopathologically confirmed infiltration of (sub peritoneal) structures and/or visceral organs, indication of an ENZIAN score by the surgeon, clinical examination findings with evidence of DE, and/or naming of procedures necessary for resection of DE procedures in the surgery report.

A questionnaire was designed to investigate the postoperative clinical course in terms of complaints, drug treatment, complementary therapies, fertility, and re-operations. The questionnaire was sent to 455 patients with a request for participation. Data were extracted from clinical reports and completed questionnaires. All patients were pseudonymized.

Approval was obtained from the institutional ethics committee of the Medical Faculty of the University of Tuebingen (400/2019BO2).

For statistical analyses and graphs R, version 3.5.1. was used. Baseline characteristics were described by median, range, mean, and standard deviation (SD) or frequencies and proportions. The reverse Kaplan–Meier method was performed for median follow-up time. Age differences were assessed by *t*-test. Furthermore, Fisher’s exact test was used for the analysis of the improvement of symptoms and fertility, depending on resection status. A significance level of 5% was chosen.

## 3. Results

### 3.1. Study Cohort

A total of 455 patients were identified for study inclusion. Within a period of three months, 133/455 (29.2%) patients responded. Due to the withdrawal of consent and an incomplete questionnaire, two patients had to be excluded. A total of 131 patients were eligible for further analyses. The median time of follow-up was 6.9 years (95% CI [6.3 years; 8.1 years]).

### 3.2. Patients’ Characteristics

The median age of the 131 patients was 34.8 years (range 16.7–49.5 years) at the time of index surgery, with a mean age of 35.0 years (SD 6.4 years). The median body mass index (BMI) was 22.7 kg/m^2^ (range 17.7–36.8 kg/m^2^, six values missing), and the mean BMI was 23.5 kg/m^2^ (SD 3.8 kg/m^2^).

### 3.3. Clinical Symptoms

**Figure 1:**
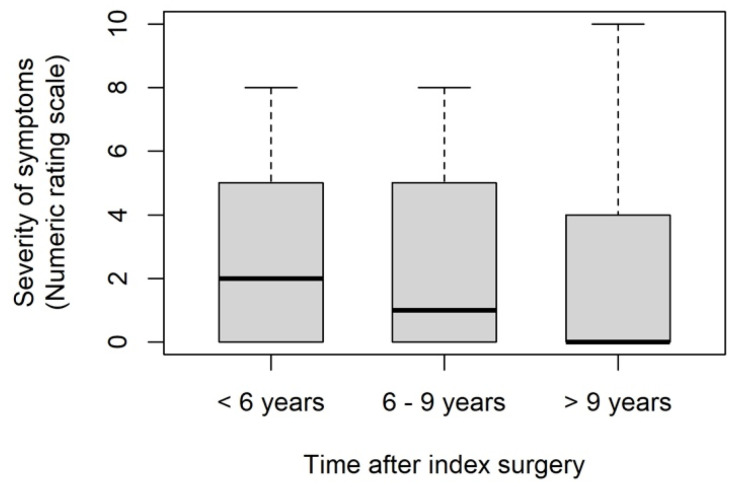
The severity of the patient’s symptoms at the time of answering the questionnaire according to follow-up time after index surgery (*p* = 0.776), recorded on a numeric rating scale (NRS): 0—no symptoms; 10—heaviest symptoms.

Fifty-eight patients (44.6%, one answer missing) stated that they were free of symptoms, whereas seventy-two patients (55.4%) still suffered from DE-related symptoms at the time of answering the questionnaire. The severity of complaints was assessed using a numerical rating scale (NRS). The answers ranged from 0 to 10 (Figure 1), while 49 of the 72 patients with residual symptoms at the time the patients answered the questionnaire (70.0%, two answers missing) chose a value between 1 and 5. A total of 107 of all patients (87.0%, eight answers missing) reported an improvement of their symptoms after the index surgery, 13 patients (10.6%) had no change, and only 3 patients (2.4%) described a deterioration of their symptoms postoperatively. There was no statistically significant difference in pain symptoms according to follow-up time (*p* = 0.776).

### 3.4. Surgical and Medical Treatment

All patients underwent surgery for treatment of DE. Laparoscopic approach was performed in 93.1%. To achieve resection of DE, ureterolysis was necessary in 114 (87.0%) cases. Furthermore, in more than one-third of cases, bowel interventions such as shaving (*n* = 24, 18.3%), disc resection (*n* = 5, 3.8%), or segment resection (*n* = 17, 13.0%) were performed. Complete resection was reached in 113 (86.3%) of all cases. An improvement in clinical symptoms was reported by 81.4% (*n* = 92) of completely resected and 83.3% (*n* = 15) of patients with residual disease after index surgery, respectively (*p* = 1.000).

Postoperative complications occurred in a total of 16 (12.2%) cases, with only one case requiring surgical revision.

Eighty-six patients (66.7%, two answers missing) reported taking medication for recurrence prophylaxis after their index surgery, and forty-three patients (33.3%) did not take any medication. Forty-eight (57.8%, three answers missing) patients taking recurrence prophylaxis experienced medication side effects. These side effects led to discontinuation of therapy in 27 (57.4%, one answer missing) cases.

### 3.5. Complementary Therapies

A total of 14 of all 131 patients (10.7%) reported receiving specific pain therapy recommended by a pain clinic after their index surgery. Forty-eight patients (36.6%) reported using additional therapies such as yoga or psychotherapy after index surgery. Thirty-seven patients (28.5%, one answer missing) reported that they underwent a rehabilitation program after their DE surgery.

### 3.6. Fertility

Infertility was described as the main indication for surgery in 41 (31.8%, two answers missing) cases. Furthermore, 66 patients (52.0%, four answers missing) reported that they tried to become pregnant after the index surgery, of which 37 (57.8%, two answers missing) were successful. Among patients who tried to become pregnant after their index surgery, 39 women (60.0%, one answer missing) used fertility treatment. A total of 17/20 patients (85.0%) who underwent fertility treatment and gave birth after their index surgery stated that fertility treatment was responsible for the realization of their desire to have a child. Looking only at the 37 patients who underwent index surgery due to unfulfilled fertility desire and subsequently attempted to become pregnant, 17 of these women (45.9%) gave birth. In contrast, 73.1% of the patients who did not undergo index surgery because of an unfulfilled fertility desire but attempted to become pregnant afterward gave birth.

Among the patients who were able to conceive, 12 women (37.5%) gave birth to one child, 19 women (59.4%) gave birth to a second child, and one woman (3.1%) gave birth to three children. There was no statistically significant difference between complete and non-complete resected patients (56.7% vs. 50.0%, *p* = 0.371)

The median time to the birth of the first child after the index surgery was 1.5 years (range 0.8–6.7 years).

### 3.7. Re-Operations

Forty-four patients (34.1%, two answers missing) reported that they had undergone re-operation for DE symptoms after their index surgery. The majority of these patients underwent additional surgery once (range 1–9).

**Figure 2:**
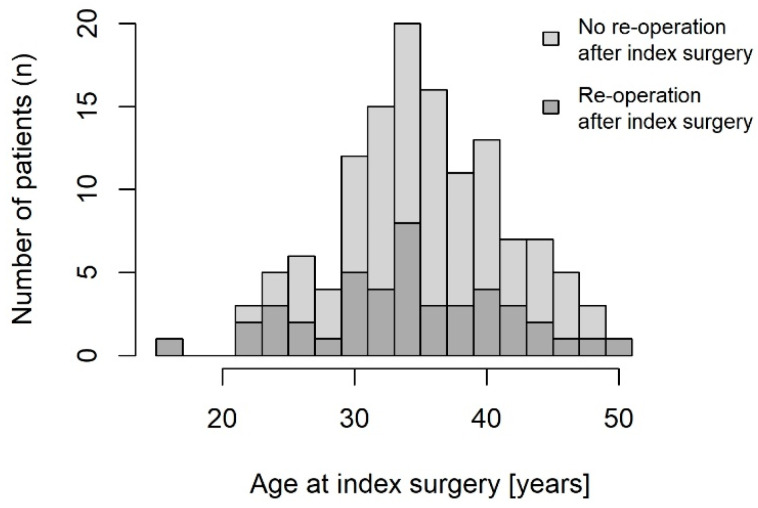
Need for re-operation due to endometriosis-typical symptoms depending on age at index surgery.

Although not statistically significant, patients with the need for another endometriosis surgery tend to be younger. The mean age of these patients at the time of index surgery was 34.1 years (SD 7.2 years) and 35.6 years (SD 5.9 years) for those without the need for re-operation, respectively (*p* = 0.226). Figure 2 shows the need for re-operation as a function of age at index surgery.

The median time from index surgery to the first re-operation due to DE symptoms was 2.1 years (range 0.2–10.9 years).

The main reason for additional surgery was pain (82.6%), and the second most common reason was an unfulfilled desire to have children (28.3%), as multiple selection was possible. The majority of re-operations (i.e., 84.2% of first re-operations) were again performed at the Women’s Health Unit Tuebingen (highest level endometriosis center).

**Figure A1:**
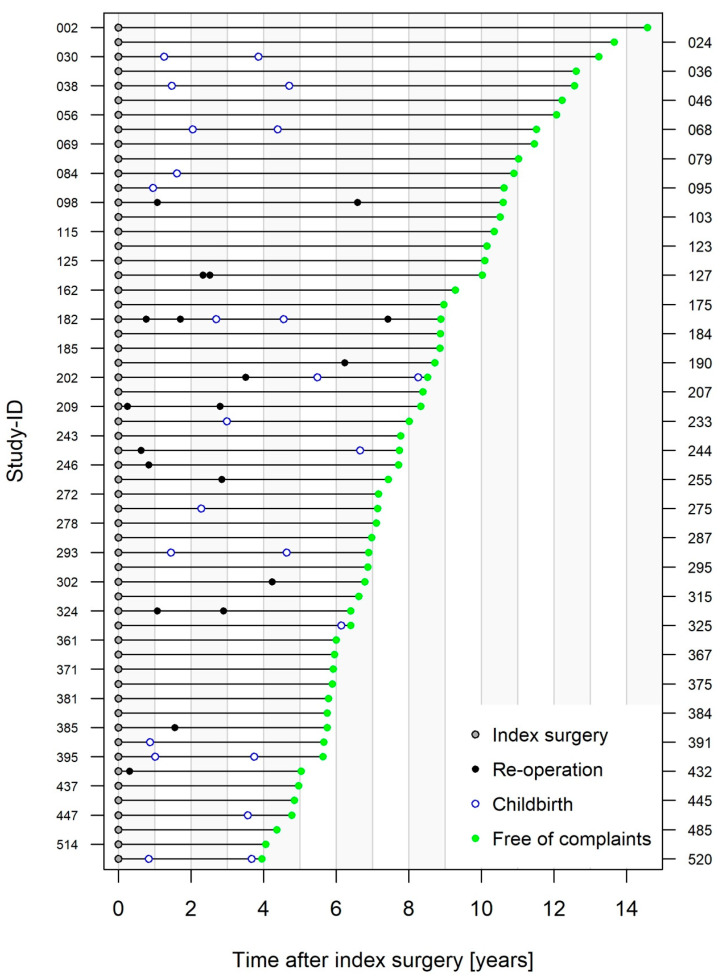
Overview of the postoperative clinical course of patients who reported no complaints at the time of answering the questionnaire.

**Figure A2:**
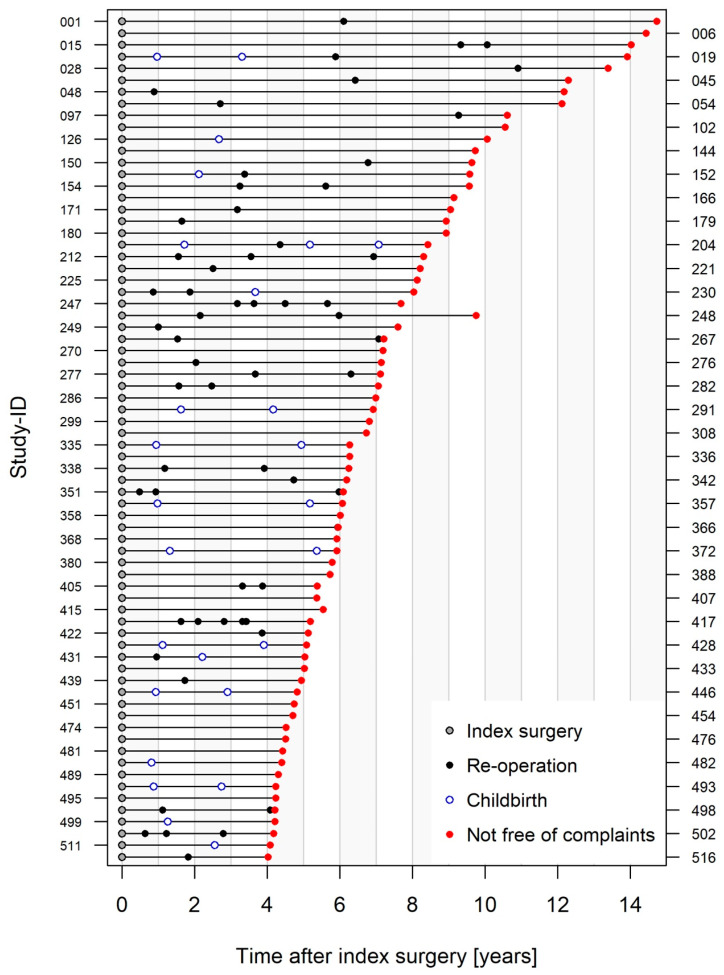
Overview of the postoperative clinical course of patients who reported still having symptoms at the time of answering the questionnaire.

Figure A1 and Figure A2 (Appendix A) provide an overview of the main parameters collected (complaints, follow-up time, re-operation, childbirth). For the sake of clarity, two graphs were created, distinguishing between patients who declared themselves to be free of complaints at the time of answering the questionnaire (Figure A1) and those who did not (Figure A2). Table A1 (Appendix B) summarizes the main parameters.

## 4. Discussion

In this retrospective study, we analyzed the clinical course of 131 patients who underwent surgery for DE.

Due to the extraordinarily long time between the index surgery (2005–2015) and the questionnaire response (2019), we aimed to evaluate in which way a substantial follow-up is possible. Thus, we focused on a broad range of recalled facts to obtain an overview of the most important parameters and to increase the data quality. Although a more detailed survey would have been desirable in some topics (i.e., medical treatment), it motivates us to investigate this in further studies.

We are pleased that we had a response rate of almost 30%. In light of the age structure of endometriosis patients with often increased mobility at this stage of life and thus difficulties in accessibility years after the index surgery, this must be seen as a remarkable percentage. Furthermore, the Women’s Health Unit Tuebingen, as the highest-level endometriosis center, treats patients all over the country with a correspondingly more difficult follow-up.

Fortunately, nearly 90% of patients reported an improvement in their symptoms after the index surgery. In addition, approximately 45% of patients reported being completely free of symptoms at the time of the survey. In our study, complete resection of endometriosis was not associated with a higher improvement in clinical symptoms. These findings emphasize the need for a symptom-orientated approach and detailed preoperative discussion with the patients about how extensive and, therefore, riskier an endometriosis surgery should be planned and performed. This marks a clear difference to oncological interventions, where complete resection is mostly mandatory for curative intention.

Similarly encouraging results were found in comparable studies [26,27]. This again underlines the value of surgical treatment of symptomatic DE, even if these surgeries are very complex. For this reason, patients should be referred to the highest-level endometriosis centers for the best expertise and results.

Although several studies could confirm the positive effects of hormonal treatment of DE after surgery to prevent the recurrence of the disease, one out of three patients in our study did not receive any medical treatment of DE after index surgery [28,29]. One reason for not taking medication could be, for example, an attempt to become pregnant immediately after surgery. Furthermore, more than half of patients using medical treatment suffered from side effects, and almost six out of ten discontinued therapy as a result.

Thus, the benefits of taking medication to reduce the risk of recurrence are weighed against the disadvantages of side effects that may lead to discontinuation of therapy. Considering this, it should be discussed whether precise information about potential side effects and how to deal with them appropriately before starting medical treatment can strengthen adherence to therapy and thus exploit the positive effects of drug therapy over a longer period of time in order to improve the patient’s quality of life and reduce the rate of re-operations.

When examining complementary therapies for endometriosis, our study found that they were not used by the majority of patients. The question arises as to whether a lack of knowledge about what is available is at least partly responsible for this. If this is the case, it is important to point out the other therapeutic options during standard therapy and, in particular, to offer establishing contact with patient groups. Getting in touch with other patients suffering from the same diagnosis might help to process negative aspects and to learn from each other coping symptoms and side effects of therapy.

In almost every third case in our study, an unfulfilled desire for a child was the reason for the endometriosis surgery. As almost one in two of these patients gave birth postoperatively, our data underscore the importance and positive effect of experienced surgical treatment as offered by the Women’s Health Unit Tuebingen. These data give hope to all DE patients suffering from infertility. Furthermore, there was no difference in the postoperative rate of pregnancy between complete and non-complete resected patients. Due to the small number of cases, more studies are needed to further investigate these findings.

Regardless of whether or not the index surgery was performed for an unfulfilled desire to have children, slightly more than half of the patients reported that they tried to become pregnant after the index surgery. Almost 60% of them gave birth to at least one child. Compared to other studies, these values can be considered pretty good, although a direct comparison does not always seem to be possible. Other studies, for example, investigated the influence of a specific surgical method in a specific subtype of DE or sometimes only determined the pregnancy rate but not the actual live birth rate [26,27,30,31].

Six out of ten patients who tried to become pregnant after the index surgery used fertility treatment. Our findings offer hope to all couples with unfulfilled desires for children that surgical treatment of endometriosis can improve fertility.

Although the index surgery led to an improvement of clinical symptoms in many patients, about every third woman in our study underwent at least one further surgery for endometriosis-typical symptoms, which underscores the chronic nature of the disease.

In addition, the mean age at index surgery of patients who underwent re-operation due to endometriosis symptoms has shown to be 1.5 years lower than that of patients who did not undergo any re-operation, even if it is not statistically significant, and the length of follow-up differs. Nevertheless, this supports the findings of other studies describing lower age as a risk factor for the recurrence of disease [20,32]. However, some studies did not report re-operation rates but rather recurrence rates, which were sometimes defined differently, making comparisons difficult [27,30,32].

Compared to other studies, the follow-up time in our study can be considered exceptionally long, with almost 15 years in some cases. For example, Donnez et al. followed up on patients who had undergone surgery for deep rectovaginal endometriotic nodules using the shaving technique for a maximum of 6 years after surgery. The recurrence rate has been shown to be 8% [30]. The differences in our study can be explained by the different follow-up times and the greater heterogeneity regarding the treated forms of DE. Therefore, a more challenging patient population can be assumed in our study.

In 2003, Abbott et al. followed patients with all forms of endometriosis for up to five years after surgery. Here, 36% required re-operation, although the follow-up period was much shorter [31]. This at least suggests that surgical techniques have improved over the years and that other effective therapeutic concepts, such as optimized medical therapy, have contributed to an improvement in the treatment of patients suffering from DE.

At the same time, it should be kept in mind that the return of symptoms does not always have to be associated with a recurrence of endometriosis but that a multifactorial process must be considered [29,31,33].

In this context, it seems essential, especially in patients with a recurrent course of the disease, to talk realistically about the goals and possibilities of therapy and to make clear that complete freedom from pain may not be achievable in some situations but that the goal of therapy is an improvement of the current complaints.

All these findings underline both the efficacy and the limitations of surgery for DE.

The results of our study must be interpreted with some limitations. First, the study is of a retrospective nature at a single institution, which carries a risk of bias. Furthermore, we used a self-administered and, therefore, non-standardized questionnaire that lacked validation. In addition, a more detailed analysis (e.g., number of fertility treatments or duration of medical treatment, including the exact type) was not possible due to the study design and the extraordinarily long follow-up period. Prospective studies are needed for more differentiated data collection.

In addition, because of the wide heterogeneity of the presentation of DE, an individualized surgical approach is required in each case, making comparability difficult.

Nevertheless, our study provides a valuable overview of the clinical course of patients who have undergone surgery for DE at a highest-level endometriosis center.

## 5. Conclusions

In this very long follow-up with a high response rate, we were able to show that surgical treatment of DE at the highest-level endometriosis center with a symptom-orientated approach based on precise knowledge about the patients’ complaints and multidisciplinary treatment improves symptoms in the majority of patients, in many cases for a long time, and can also substantially improve fertility. If medical recurrence prophylaxis is used after surgery, potential side effects, which may even force some patients to discontinue therapy, should be considered. Complementary therapies are not regularly used, so it may be helpful to point out their availability. Despite all the positive aspects, there is still a considerable risk of recurrence in DE, with the subsequent need for further surgery.

## Data Availability

Data are available upon request from the corresponding author.

## Appendix A

Please note that the follow-up time of the patient with study ID 248 is visible as an outlier. This is due to the fact that after the study IDs were assigned consecutively after the first database query, a second look revealed that another surgery for DE had taken place before, but still within the survey period. Therefore, the date of the index surgery had to be adjusted.

## Appendix B

**Table A1:** Overview of the patients’ baseline characteristics and main follow-up parameters after index surgery.

VARIABLES	
**Patients’ characteristics**	
Age at index surgery, years ^a^	35.0 (6.4) [16.7–49.5]
Body mass index (BMI) at index surgery, kg/m^2 a^	23.5 (3.8) [17.7–36.8]
Follow-up time, years ^b^	6.9 [6.3; 8.1]
**Complaints**	
Complaints at the time of answering the questionnaire, *n*	
Yes	72 (55.4%)
No	58 (44.6%)
Change of complaints after the index surgery, *n*	
Better	107 (87.0%)
Same	13 (10.6%)
Worse	3 (2.4%)
**Medical treatment**	
Medication intake for recurrence prophylaxis after index surgery, *n*	
Yes	86 (66.7%)
No	43 (33.3%)
Side effects due to medication intake, *n*	
Yes	48 (57.8%)
No	35 (42.2%)
Discontinuation of medication intake due to side effects, *n*	
Yes	27 (57.4%)
No	20 (42.6%)
**Complementary therapies**	
Specific pain therapy after index surgery, *n*	
Yes	14 (10.7%)
No	117 (89.3%)
Additional therapies (yoga, psychotherapy etc.), *n*	
Yes	48 (36.6%)
No	83 (63.4%)
Rehabilitation, *n*	
Yes	37 (28.5%)
No	93 (71.5%)
**Fertility**	
Index surgery due to unfulfilled desire to have a child, *n*	
Yes	41 (31.8%)
No	88 (68.2%)
Try to become pregnant after index surgery, *n*	
Yes	66 (52.0%)
No	61 (48.0%)
Childbirth after index surgery, *n*	
Yes	37 (57.8%)
No	27 (42.2%)
Time to first childbirth after index surgery, years ^c^	1.5 [0.8–6.7]
Use of fertility treatment, *n*	
Yes	39 (60.0%)
No	26 (40.0%)
**Re-operations**	
Need for re-operation due to endometriosis-typical complaints, *n*	
Yes	44 (34.1%)
No	85 (65.9%)
Time to first re-operation after index surgery, years ^c^	2.1 [0.2–10.9]

^a^ Data are characterized as mean (SD) and range; ^b^ data are characterized as median and 95% confidence interval; ^c^ data are characterized as median and range.
